# Specifying the Unitary Evolution of a Qudit for a General Nonstationary Hamiltonian via the Generalized Gell-Mann Representation

**DOI:** 10.3390/e22050521

**Published:** 2020-05-03

**Authors:** Elena R. Loubenets, Christian Käding

**Affiliations:** 1 Applied Mathematics Department, National Research University Higher School of Economics, Moscow 101000, Russia; kkaeding@hse.ru; 2 Steklov Mathematical Institute of Russian Academy of Sciences, Moscow 119991, Russia

**Keywords:** unitary evolution of a qudit, nonstationary Hamiltonian, exponential representation, Bloch-like vector space, analytical solutions

## Abstract

Optimal realizations of quantum technology tasks lead to the necessity of a detailed analytical study of the behavior of a *d*-level quantum system (qudit) under a time-dependent Hamiltonian. In the present article, we introduce a new general formalism describing the unitary evolution of a qudit (d≥2) in terms of the Bloch-like vector space and specify how, in a general case, this formalism is related to finding time-dependent parameters in the exponential representation of the evolution operator under an arbitrary time-dependent Hamiltonian. Applying this new general formalism to a qubit case (d=2), we specify the unitary evolution of a qubit via the evolution of a unit vector in $R^{4}$, and this allows us to derive the precise analytical expression of the qubit unitary evolution operator for a wide class of nonstationary Hamiltonians. This new analytical expression includes the qubit solutions known in the literature only as particular cases.

## 1. Introduction

Optimal realizations of many quantum technology tasks need a detailed analysis of the evolution of a d≥2 dimensional quantum system (a qudit) under a time-dependent Hamiltonian H(t). In mathematical terms, the evolution of a qudit under a Hamiltonian H(t) is described on the complex Hilbert space $C^{d}$ by the unitary operator $U_{H}\left(t,t_{0}\right)$—the solution of the Cauchy problem for the nonstationary Schrödinger equation with the initial condition $U_{H}\left(t_{0},t_{0}\right)=I$. For a time-independent Hamiltonian *H*, the solution of this Cauchy problem is well-known and reads $U_{H}\left(t,t_{0}\right)=\exp\left\{-iH(t-t_{0})\right\}$.

If a Hamiltonian H(t) depends on time, then $U_{H}\left(t,t_{0}\right)$ is formally given by the *T*-chronological exponent [1,2]—the infinite Volterra series (see Equation (4) in Section 2)—which however converges only under some suitable conditions on H(t). For some nonstationary Hamiltonians beyond these conditions, the analytical expressions for $U_{H}\left(t,t_{0}\right)$ via parameters of H(t) are also known, for example, for a free electron [3] in a magnetic field spinning around the $x_{3}$-axis. However, for an arbitrary time-dependent H(t), the analytical expression for $U_{H}\left(t,t_{0}\right)$ via parameters of H(t) is not known even in a qubit case.

On the other hand, every unitary operator *V* on the complex Hilbert space $C^{d}$ has the form $\exp\left\{-i\alpha \right\}\tilde{V},$α∈R, where a unitary operator $\tilde{V}$ is an element of the SU(d) group and, hence, admits the exponential parametrization via the SU(d) group generators. Therefore, for a *d*-dimensional quantum system, the exponential representation for $U_{H}\left(t,t_{0}\right)$ must also exist and there arises the problem of how to determine time-dependent parameters of this exponential representation via characteristics of a given qudit Hamiltonian H(t). To our knowledge, the solution of this problem has not been reported in the literature even for a qubit case.

In this article, we introduce a new general formalism describing the unitary evolution of a qudit (d≥2) in terms of the Bloch-like vector space and specify how in a general case this formalism is related to finding time-dependent parameters in the exponential representation of $U_{H}\left(t,t_{0}\right)$ under an arbitrary time-dependent Hamiltonian.

Applying this general formalism to a qubit case (d=2), we specify the unitary evolution of a qubit via the evolution of a unit vector in $R^{4}$ and find the precise analytical expression of $U_{H}\left(t,t_{0}\right)$ for a wide class of nonstationary qubit Hamiltonians. This new analytical expression includes the qubit solutions known in the literature only as particular cases.

The article is organized as follows.

In Section 2, we analyze the known representations for $U_{H}\left(t,t_{0}\right)$ and discuss the properties of the generalized Gell-Mann representation for an arbitrary Hamiltonian and an arbitrary unitary operator on $C^{d}$ (different aspects of the Bloch-like representations for qudits were considered in References [4,5,6,7,8,9,10,11]).

In Section 3, we derive (Theorem 1) the new general equations specifying the unitary evolution of a qudit (d≥2) under a Hamiltonian H(t) in terms of parameters in the generalized Gell-Mann representation and in the exponential representation of $U_{H}\left(t,t_{0}\right).$

In Section 4 and Section 5, we specify (Theorem 2) the forms of these new general equations in a qubit case (d=2) and derive the novel precise analytical expression of $U_{H}\left(t,t_{0}\right)$ for a wide class of qubit Hamiltonians H(t).

The main results of the article are summarized in Section 6.

## 2. Unitary Evolution of a Qudit (d≥2)

Let H(t):$C^{d}\to C^{d},$$H\left(t\right)=H^{†}\left(t\right),$ d≥2, be a Hamiltonian of a *d*-level quantum system (qudit). The evolution of a qudit state

(1) $$\rho \left(t\right)=U_{H}\left(t,t_{0}\right)\rho \left(t_{0}\right)U_{H}^{†}\left(t,t_{0}\right),\;\;\;t\geq t_{0},$$

under a Hamiltonian H(t) is determined by the unitary operator $U_{H}\left(t,t_{0}\right)$—the solution of the Cauchy problem for the nonstationary Schrödinger equation

(2) $$\begin{array}{cc} i\frac{d}{dt}U_{H}\left(t,t_{0}\right) & =H\left(t\right)U_{H}\left(t,t_{0}\right),\;\;\;t>t_{0},\\ U_{H}\left(t_{0},t_{0}\right) & =I, \end{array}$$

which satisfies the cocycle property

(3) $$U_{H}\left(t,t_{0}\right)=U_{H}\left(t,s\right)U_{H}\left(s,t_{0}\right),\;\;\;s\in \left[t,t_{0}\right],$$

and is represented by the chronological operator exponent

(4) $$\begin{array}{cc} U_{H}\left(t,t_{0}\right) & =T\exp\left\{-i\int_{t_{0}}^{t}H\left(\tau \right)d\tau \right\}\\ & =I-i\int_{t_{0}}^{t}H\left(\tau \right)d\tau +\frac{1}{2}{\left(-i\right)}^{2}\int_{t_{0}}^{t}d\tau_{1}\int_{t_{0}}^{t}d\tau_{2}T\left\{H\left(\tau_{1}\right)H\left(\tau_{2}\right)\right\}+\ldots \\ & +\frac{1}{n!}{\left(-i\right)}^{n}\int_{t_{0}}^{t}d\tau_{1}\int_{t_{0}}^{t}d\tau_{2}T\left\{\;H\left(\tau_{1}\right)H\left(\tau_{2}\right)\cdot \ldots \cdot H\left(\tau_{n}\right)\right\}+\ldots , \end{array}$$

where symbol T{·} means

(5) $$T\left\{H\left(\tau_{1}\right)\cdot \ldots \cdot H\left(\tau_{m}\right)\right\}:=H\left(\tau_{\alpha_{1}}\right)\cdot \ldots \cdot H\left(\tau_{\alpha_{m}}\right),\;\;\;\tau_{\alpha_{1}}\geq \tau_{\alpha_{1}}>\ldots >\tau_{\alpha_{m}}.$$

If a Hamiltonian H(t) satisfies the condition

(6) $$\left[H\left(t\right),\int_{t_{0}}^{t}H\left(\tau \right)d\tau \right]=0,\;\;t>t_{0},\;$$

then the series in Equation (2) reduces to

(7) $$U_{H}\left(t,t_{0}\right)=\exp\left\{-i\int_{t_{0}}^{t}H\left(\tau \right)d\tau \right\}.$$

Recall (see, for example, References [4,5,6,7,8]) that any linear operator *A* on $C^{d}$ admits the representation via the generalized Gell-Mann matrices—the generalized Gell-Mann representation:

(8) $$\begin{array}{cc} A & =a_{0}\;I\;\;+\;\;\sqrt{\frac{d}{2}}\;a\cdot \Lambda ,\;\;\;\;a\cdot \Lambda :=\sum_{j=1,\ldots ,d^{2}-1}a_{j}\cdot \Lambda_{j},\\ a_{0} & =\frac{1}{d}\mathrm{tr}\left[A\right]\in C,\;\;\;a_{j}=\frac{1}{\sqrt{2d}}\mathrm{tr}\left[A\Lambda_{j}\right]\in C,\;\;\;a=\left(a_{1},\ldots a_{d^{2}-1}\right), \end{array}$$

where $\Lambda =(\Lambda_{1},\ldots \Lambda_{d^{2}-1})$ is a tuple of traceless Hermitian operators on $C^{d}$:

(9) $$\Lambda_{k}=\Lambda_{k}^{†},\;\;\;\mathrm{tr}\left[\Lambda_{k}\right]=0,\;\;\;k=1,\ldots ,\left(d^{2}-1\right),$$

satisfying the relations

(10) $$\begin{array}{cc} \Lambda_{k}\Lambda_{m} & =\frac{2}{d}\delta_{km}\;I\;+\;\sum_{j}\left(d_{kmj}+if_{kmj}\right)\Lambda_{j},\\ {}[\Lambda_{k},\Lambda_{m}] & =2i\sum_{j}f_{kmj}\Lambda_{j},\;\;\;\;\mathrm{tr}\left[\Lambda_{k}\Lambda_{m}\right]=2\delta_{km}, \end{array}$$

and constituting generators of group SU(d). In Equation (10), $\delta_{km}$ is the Kronecker symbol and $f_{jkm}$, $d_{jkm}$ are antisymmetric and symmetric structure coefficients of SU(d), respectively. The matrix representations of the operators $\Lambda_{j},$ $j=1,\ldots ,(d^{2}-1),$ in the computational basis of $C^{d}$ constitute the higher-dimensional extensions of the Pauli matrices in the qubit case (d=2) and the Gell-Mann matrices in the qutrit case (d=3).

For a vector *a* in Equation (8)

(11) $$\mathrm{tr}\left[A^{†}A\right]=d\left({\left|a_{0}\right|}^{2}+{∥a^{\prime }∥}_{C^{d^{2}-1}}^{2}\right),$$

where we choose the same normalization of a vector *a* in representation (8) as for traceless qudit observables in Reference [8]. Here and in what follows, by the upper prime $r^{\prime }\in C^{d^{2}-1}$, we denote the column-vector comprised of components of a vector $r=(r_{1},\ldots ,r_{d^{2}-1})$.

Note that representation (8) constitutes the decomposition of a linear operator *A* on $C^{d}$ in the orthogonal basis

(12) $$\left\{\;I,\Lambda_{1},\ldots ,\Lambda_{d^{2}-1}\right\}$$

of the vector space L where linear operators $A:C^{d}\to C^{d}$ constitute vectors, and the scalar product is defined by ${\langle A_{1},A_{2}\rangle }_{L}:=\mathrm{tr}\left[A_{1}^{†}A_{2}\right].$

For a Hamiltonian H(t) on $C^{d},$ the generalized Gell-Mann representation (8) reads

(13) $$\begin{array}{ccc} H(t) & = & b_{0}\left(t\right)I\;\;+\;\;\sqrt{\frac{d}{2}}\left(b_{H}\left(t\right)\cdot \Lambda \right),\\ b_{0}\left(t\right) & = & \frac{1}{d}\mathrm{tr}\left[H\left(t\right)\right]\in R,\;\;\;b_{H}^{\left(j\right)}\left(t\right)=\frac{1}{\sqrt{2d}}\mathrm{tr}\left[H\left(t\right)\Lambda_{j}\right]\in R,\\ b_{H}\left(t\right) & = & \left(b_{H}^{\left(1\right)}\left(t\right),\ldots ,b_{H}^{\left(d^{2}-1\right)}\left(t\right)\right)\in R^{d^{2}-1}, \end{array}$$

and condition (6) implies the following limitations on a vector $b_{H}\left(t\right)\in R^{d^{2}-1}$:

(14) $$\sum_{k,m}f_{kmj}b_{H}^{\left(k\right)}\left(t\right)\left(\int_{t_{0}}^{t}b_{H}^{\left(m\right)}\left(\tau \right)d\tau \right)=0,\;\;\;j=1,\ldots ,\left(d^{2}-1\right).$$

Therefore, if a vector $b_{H}\left(t\right)$ satisfies conditions (14), then, by Equation (7),

(15) $$U_{H}\left(t,t_{0}\right)=\exp\left\{-i\int_{t_{0}}^{t}b_{0}\left(\tau \right)d\tau \right\}\exp\left\{-i\sqrt{\frac{d}{2}}\left(\int_{t_{0}}^{t}b_{H}\left(\tau \right)d\tau \right)\cdot \Lambda \right\}.$$

However, for an arbitrary qudit Hamiltonian H(t), condition (6) (equivalently, condition (14)) does not need to be fulfilled, so that the exponential representation (15) of $U_{H}\left(t,t_{0}\right)$ via the decomposition coefficients $b_{0}\left(t\right),$$b_{H}\left(t\right)$ of a Hamiltonian H(t) by Equation (13) does not, in general, hold.

On the other hand, as it is the case for every unitary operator on $C^{d},$ operator $U_{H}\left(t,t_{0}\right)$ must have the form

(16) $$U_{H}\left(t,t_{0}\right)=\exp\left\{-i\alpha (t,t_{0})\right\}{\tilde{U}}_{H}\left(t,t_{0}\right),\;\;\;\alpha \left(t,t_{0}\right)\in R,$$

where ${\tilde{U}}_{H}\left(t,t_{0}\right)\in \mathrm{SU}\left(d\right)$ and, hence, as any element of SU(d), admits (see, for example, Reference [12] and references therein) the exponential parametrization

(17) $${\tilde{U}}_{H}\left(t,t_{0}\right)=\exp\left\{-i\;\sqrt{\frac{d}{2}}\left(n_{H}\left(t,t_{0}\right)\cdot \Lambda \right)\right\}$$

via generators $\Lambda_{1},\ldots \Lambda_{d^{2}-1}$ of group SU(d) and a vector $n_{H}\left(t,t_{0}\right)=\left(n_{1},\ldots n_{d^{2}-1}\right)\in R^{d^{2}-1},$ which in case of solution ${\tilde{U}}_{H}\left(t,t_{0}\right)\in \mathrm{SU}\left(d\right)$ depends also on a Hamiltonian H(t), time *t* and an initial moment $t_{0}$. In Equation (17), similarly as in decomposition (8), we use the following normalization for a vector $n_{H}\left(t,t_{0}\right):$

(18) $$\mathrm{tr}\left[{\left(\sqrt{\frac{d}{2}}n_{H}\left(t,t_{0}\right)\cdot \Lambda \right)}^{2}\right]=d{∥n_{H}\left(t,t_{0}\right)∥}_{R^{d^{2}-1}}^{2}.$$

Relations (16) and (17) imply that, for every qudit Hamiltonian H(t), for which a unique solution of Equation (2) exists, the unitary evolution operator $U_{H}\left(t,t_{0}\right)$ admits the exponential representation

(19) $$\begin{array}{cc} U_{H}\left(t,t_{0}\right) & =\exp\left\{-i\alpha (t,t_{0})\right\}\exp\left\{-i\;\sqrt{\frac{d}{2}}\left(n_{H}\left(t,t_{0}\right)\cdot \Lambda \right)\right\},\\ \alpha (t_{0},t_{0}) & =0,\;\;\;n_{H}\left(t_{0},t_{0}\right)=0, \end{array}$$

where parameters $\alpha (t,t_{0}),$$n_{H}\left(t,t_{0}\right)$ can be presented in the form

(20) $$\alpha \left(t,t_{0}\right)=\int_{t_{0}}^{t}\beta_{0}\left(\tau \right)d\tau ,\;\;\;\beta_{0}\left(\tau \right)\in R,\;\;\;n_{H}\left(t,t_{0}\right)=\int_{t_{0}}^{t}\beta_{H}\left(\tau \right)d\tau ,\;\;\;\beta_{H}\left(t\right)\in R^{d^{2}-1}.$$

This implies

(21) $$U_{H}\left(t,t_{0}\right)=\exp\left\{-i\int_{t_{0}}^{t}\beta_{0}\left(\tau \right)d\tau \right\}\exp\left\{-i\;\sqrt{\frac{d}{2}}\left(\int_{t_{0}}^{t}\beta_{H}\left(\tau \right)d\tau \right)\cdot \Lambda \right\}.$$

The form of this representation is quite similar to the one of representation (15), which is valid if a Hamiltonian H(t) satisfies condition (14). However, for an arbitrary Hamiltonian H(t), a vector $\beta_{H}\left(t\right)\in R^{d^{2}-1}$ in Equation (21) does not need to be equal to a vector $b_{H}\left(t\right)\in R^{d^{2}-1}$ in representation (13) for this H(t).

Therefore, in order to specify the unitary evolution operator $U_{H}\left(t,t_{0}\right)$ under an arbitrary nonstationary Hamiltonian H(t), we need to express parameters $\beta_{0}\left(t\right)\in R,$$\beta_{H}\left(t\right)\in R^{d^{2}-1}$ in Equation (21) via coefficients $b_{0}\left(t\right)\in R,$$b_{H}\left(t\right)\in R^{d^{2}-1}$ in the generalized Gell-Mann representation (13) for a given H(t).

In the proceeding sections, we consider this problem for an arbitrary d≥2 and further study the case d=2 in detail.

## 3. Evolution Equations in the Bloch-Like Vector Space

Together with the generalized Gell-Mann representation (13) for a Hamiltonian H(t), let us also specify decomposition (8) for a unitary operator (17) on $C^{d}$:

(22) $$\begin{array}{cc} {\tilde{U}}_{H}\left(t,t_{0}\right) & =\exp\left\{-i\;\sqrt{\frac{d}{2}}\left(n_{H}\left(t,t_{0}\right)\cdot \Lambda \right)\right\}\\ \; & =u_{0}\left(t,t_{0}\right)I\;+\;\sqrt{\frac{d}{2}}\;u_{H}\left(t,t_{0}\right)\cdot \Lambda ,\\ u_{H}\left(t,t_{0}\right) & =(u_{H}^{\left(1\right)},\ldots ,u_{H}^{\left(d^{2}-1\right)}),\\ u_{0}\left(t,t_{0}\right) & =\frac{1}{d}\mathrm{tr}\left[{\tilde{U}}_{H}\left(t,t_{0}\right)\right]\in C,\\ u_{H}^{\left(j\right)}\left(t,t_{0}\right) & =\frac{1}{\sqrt{2d}}\mathrm{tr}\left[{\tilde{U}}_{H}\left(t,t_{0}\right)\Lambda_{j}\right]\in C. \end{array}$$

The initial conditions in Equation (19) and the unitary property of ${\tilde{U}}_{H}\left(t,t_{0}\right)$ imply

(23) $$u_{0}\left(t_{0},t_{0}\right)=1,\;\;\;u_{H}^{\left(j\right)}\left(t_{0},t_{0}\right)=0,$$

and

(24) $${\left|u_{0}\left(t,t_{0}\right)\right|}^{2}+{∥u_{H}^{{}^{\prime }}\left(t,t_{o}\right)∥}_{C^{d^{2}-1}}^{2}=1,$$

$$\begin{array}{cc} \; & u_{0}\left(t,t_{0}\right){\left(u_{{}_{H}}^{\left(j\right)}\left(t,t_{0}\right)\right)}^{*}+u_{0}^{*}\left(t,t_{0}\right)u_{H}^{\left(j\right)}\left(t,t_{0}\right)\\ \; & +\sqrt{\frac{d}{2}}\sum_{k,m}\left(d_{kmj}+if_{kmj}\right)u_{H}^{\left(k\right)}\left(t,t_{0}\right){\left(u_{H}^{\left(m\right)}\left(t,t_{0}\right)\right)}^{*}=0, \end{array}$$

for all $t\geq t_{0}$ and all $j=1,\ldots ,(d^{2}-1).$

Substituting Equation (22) into Equation (19), Equation (19) into Equation (2), and taking $u_{H}\left(t,t_{0}\right)=i{\tilde{u}}_{H}\left(t,t_{0}\right)$, we derive

(25) $$\alpha \left(t,t_{0}\right)=\exp\left\{-i\int_{t_{0}}^{t}b_{0}\left(\tau \right)d\tau \right\}$$

and the following system of linear ordinary differential equations for $u_{0}\left(t,t_{0}\right)$ and ${\tilde{u}}_{H}\left(t,t_{0}\right):$

(26) $$\begin{array}{cc} {\overset{\cdot }{u}}_{0}\left(t,t_{0}\right) & =b_{{}_{H}}\left(t\right)\cdot {\tilde{u}}_{H}\left(t,t_{0}\right),\\ \frac{d}{dt}{\tilde{u}}_{H}^{\left(j\right)}\left(t,t_{0}\right) & =-u_{0}\left(t,t_{0}\right)b_{H}^{\left(j\right)}+\sqrt{\frac{d}{2}}\sum_{m,k}\left(\;f_{kmj}-id_{kmj}\right)b_{H}^{\left(k\right)}\left(t\right){\tilde{u}}_{H}^{\left(m\right)}\left(t,t_{0}\right),\\ u_{0}\left(t_{0},t_{0}\right) & =1,\;\;\;{\tilde{u}}_{H}\left(t_{0},t_{0}\right)=0. \end{array}$$

Relation (24) constitute the functionally independent first integrals of these ordinary differential equations (ODEs).

Thus, for an arbitrary d≥2, the unitary evolution operator $U_{H}\left(t,t_{0}\right)$ under a Hamiltonian H(t) is given by

(27) $$\begin{array}{cc} U_{H}\left(t,t_{0}\right) & =\exp\left\{-i\int_{t_{0}}^{t}b_{0}\left(\tau \right)d\tau \right\}\exp\left\{-i\;\sqrt{\frac{d}{2}}\left(n_{H}\left(t,t_{0}\right)\cdot \Lambda \right)\right\}\\ \; & =\exp\left\{-i\int_{t_{0}}^{t}b_{0}\left(\tau \right)d\tau \right\}\left(u_{0}\left(t,t_{0}\right)\;I\;+\;i\;\sqrt{\frac{d}{2}}\;{\tilde{u}}_{H}\left(t,t_{0}\right)\cdot \Lambda \right), \end{array}$$

where $u_{0}\left(t,t_{0}\right)\in C,$${\tilde{u}}_{H}^{\prime }\left(t,t_{0}\right)\in C^{d^{2}-1}$ satisfy the Cauchy problem (26) for the nonautonomous system of linear ordinary differential equations (ODEs).

On the other hand, due to the results in Reference [12], we can explicitly represent $u_{0}\left(t\right)\in C,$${\tilde{u}}_{H}^{\prime }\left(t\right)\in C^{d^{2}-1}$ in Equation (27) via a vector $n_{H}\left(t,t_{0}\right)$.

Namely, for each group element $V_{d}\in \mathrm{SU}\left(d\right)$ with the exponential parametrization

(28) $$V_{d}\left(r\right)=\exp\left\{-i\sqrt{\frac{d}{2}}\left(r\cdot \Lambda \right)\right\},\;\;\;r\in R^{d^{2}-1},$$

let us consider the generalized Gell-Mann representation (8):

(29) $$\begin{array}{cc} \exp\left\{-i\sqrt{\frac{d}{2}}\left(r\cdot \Lambda \right)\right\} & =v_{0}\left(r\right)\;I\;\;+\;\;\sqrt{\frac{d}{2}}\left(v(r)\cdot \Lambda \right),\\ {\left|v_{0}\left(t\right)\right|}^{2}+{∥v^{\prime }\left(t\right)∥}_{C^{d^{2}-1}}^{2} & =1, \end{array}$$

where

(30) $$v_{0}\left(r\right)=\frac{1}{d}\mathrm{tr}\left[\exp\left\{-i\sqrt{\frac{d}{2}}\left(r\cdot \Lambda \right)\right\}\right],\;v\left(r\right)=\frac{1}{\sqrt{2d}}\mathrm{tr}\left[\Lambda \exp\left\{-i\sqrt{\frac{d}{2}}\left(r\cdot \Lambda \right)\right\}\right].$$

Denote by $E(\lambda_{m}\left(r\right))$ the spectral projection of a Hermitian operator (r·Λ) corresponding to its eigenvalue $\lambda_{m}\left(r\right)\in R$ with multiplicity $k_{\lambda_{{}_{m}}\left(r\right)}$. The spectral decomposition of $V_{d}\left(r\right)=\exp\left\{-i\sqrt{\frac{d}{2}}\left(r\cdot \Lambda \right)\right\}$ reads

(31) $$V_{d}\left(r\right)=\sum_{\lambda_{m}}\exp\left\{-i\sqrt{\frac{d}{2}}\lambda_{m}\left(r\right)\right\}E\left(\lambda_{m}\left(r\right)\right).$$

Substituting this into relations in Equation (30) and taking into account the cyclic property of the trace and relation $\mathrm{tr}\left[E\left(\lambda_{m}\left(r\right)\right)\right]=k_{\lambda_{{}_{m}}\left(r\right)}$, we derive (these expressions differ by normalizations from those in Reference [12])

(32) $$v_{0}\left(r\right)=\frac{1}{d}\mathrm{tr}\left[V_{d}\left(r\right)\right]=\frac{1}{d}\sum_{\lambda_{m}}k_{\lambda_{{}_{m}}\left(r\right)}\exp\left\{-i\sqrt{\frac{d}{2}}\lambda_{m}\left(r\right)\right\},$$

(33) $$\begin{array}{cc} v_{j}\left(r\right) & =\frac{1}{\sqrt{2d}}\mathrm{tr}\left[\Lambda_{j}V_{d}\left(r\right)\right]=\frac{1}{\sqrt{2d}}\mathrm{tr}\left[\Lambda_{j}\left(\sum_{m=0,1,\ldots }\frac{{\left(-i\right)}^{m}}{m!}{\left(\frac{d}{2}\right)}^{\frac{m}{2}}{\left(r\cdot \Lambda \right)}^{m}\right)\right]\\ \; & =\frac{i}{d}\frac{\partial }{\partial r_{j}}\mathrm{tr}\left[V_{d}\left(r\right)\right], \end{array}$$

which imply

(34) $$\begin{array}{cc} v_{0}\left(r\right) & =\frac{1}{d}K_{d}\left(r\right),\;\;\;v_{j}\left(r\right)=\frac{i}{d}\left(\nabla_{r}\;K_{d}\left(r\right)\cdot \Lambda \right),\\ V_{d}\left(r\right) & =\frac{1}{d}K_{d}\left(r\right)I\;+\;i\sqrt{\frac{d}{2}}\left(\frac{1}{d}\nabla_{r}\;K_{d}\left(r\right)\cdot \Lambda \right), \end{array}$$

where $\nabla_{r}:=\left(\frac{\partial }{\partial r_{1}},\ldots ,\frac{\partial }{\partial r_{d^{2}-1}}\right)$ and

(35) $$K_{d}\left(r\right):=\sum_{\lambda_{m}\left(r\right)}k_{\lambda_{m}\left(r\right)}\exp\left\{-i\sqrt{\frac{d}{2}}\lambda_{m}\left(r\right)\right\}.$$

From Equations (29) and (34), it follows that, in relations in Equation (27),

(36) $$\begin{array}{cc} u_{0}\left(t,t_{0}\right) & =\frac{1}{d}K_{d}\left(n_{H}\left(t,t_{0}\right)\right),\\ {\tilde{u}}_{{}_{H}}\left(t\right) & =\frac{1}{d}\nabla_{n_{{}_{H}}}\left(K_{d}(n_{H}\left(t,t_{0}\right)\right)), \end{array}$$

for some vector $n_{H}\left(t,t_{0}\right)\in R^{d^{2}-1},$ so that

(37) $$\begin{array}{cc} {\tilde{U}}_{H}\left(t,t_{0}\right) & =\exp\left\{-i\;\sqrt{\frac{d}{2}}\left(n_{H}\left(t,t_{0}\right)\cdot \Lambda \right)\right\}\\ \; & =\frac{1}{d}K_{d}\left(n_{H}\left(t,t_{0}\right)\right)\;I\;+\;i\frac{1}{\sqrt{2d}}\nabla_{n_{{}_{H}}}\left(K_{d}(n_{H}\left(t,t_{0}\right)\right))\cdot \Lambda . \end{array}$$

The substitution of Equation (36) into the first and the second equations of the system of linear ODEs of Equation (26) gives

(38) $$\begin{array}{cc} \frac{\partial }{\partial n_{H}}K_{d}\left(n_{H}\left(t,t_{0}\right)\right)\cdot \frac{dn_{H}\left(t,t_{0}\right)}{dt} & =b_{H}\left(t\right)\cdot \frac{\partial }{\partial n_{H}}K_{d}\left(n_{H}\left(t,t_{0}\right)\right)\\ \; & ⇕\\ \frac{dn_{H}\left(t,t_{0}\right)}{dt}-b_{H}\left(t\right)\;\; & ⊥\;\;\frac{\partial }{\partial n_{H}}K_{d}\left(n_{H}\left(t,t_{0}\right)\right) \end{array}$$

and

(39) $$\begin{array}{cc} \; & \frac{\partial }{\partial n_{H}}\left(\frac{\partial }{\partial n_{H}^{\left(j\right)}}\left(K_{d}(n_{H}\left(t,t_{0}\right)\right)\right)\cdot \frac{dn_{H}\left(t,t_{0}\right)}{dt}\\ \; & =-b_{H}^{\left(j\right)}\left(t\right)K_{d}\left(n_{H}\left(t,t_{0}\right)\right)\;+\;\sqrt{\frac{d}{2}}\sum_{k,m}\left(\;f_{kmj}-id_{kmj}\right)b_{H}^{\left(k\right)}\left(t\right)\frac{\partial }{\partial n_{H}^{\left(m\right)}}K_{d}\left(n_{H}\left(t,t_{0}\right)\right),\\ j & =1,\ldots ,(d^{2}-1), \end{array}$$

respectively.

Relations in Equations (19) and (22)–(39) prove the following statement.

**Theorem** **1.** *Let $H\left(t\right)=b_{0}\left(t\right)I+b\left(t\right)\cdot \Lambda ,$$b\left(t\right)\in R^{d^{2}-1},$ be a Hamiltonian on $C^{d}.$ For each d≥2, the solution of the Cauchy problem for the nonstationary Schrödinger equation (Equation (2))—the unitary operator $U_{H}\left(t,t_{0}\right)$ on $C^{d}$ describing the evolution of a qudit under a Hamiltonian H(t)—has the form*

(40) $$\begin{array}{cc} U_{H}\left(t,t_{0}\right) & =\exp\left\{-i\int_{t_{0}}^{t}b_{0}\left(\tau \right)d\tau \right\}\exp\left\{-i\;\sqrt{\frac{d}{2}}\left(n_{H}\left(t,t_{0}\right)\cdot \Lambda \right)\right\}\\ \; & =\exp\left\{-i\int_{t_{0}}^{t}b_{0}\left(\tau \right)d\tau \right\}\left(u_{0}\left(t,t_{0}\right)\;I\;+\;i\sqrt{\frac{d}{2}}\;{\tilde{u}}_{{}_{H}}\left(t,t_{0}\right)\cdot \Lambda \right). \end{array}$$

*Here, the scalar function $u_{0}\left(t,t_{0}\right)\in C$ and vector $u_{H}\left(t,t_{0}\right)=\left(u_{H}^{\left(1\right)},\ldots ,u_{H}^{\left(d^{2}-1\right)}\right),$$u_{H}^{\left(j\right)}\in C,$ are the solutions of the Cauchy problem in Equation (26), equivalently,*

(41) $$u_{0}\left(t,t_{0}\right)=\frac{1}{d}K_{d}\left(n_{H}\left(t,t_{0}\right)\right),\;\;\;{\tilde{u}}_{H}\left(t,t_{0}\right)=\frac{1}{d}\nabla_{n_{{}_{H}}}\left(K_{d}(n_{H}\left(t,t_{0}\right)\right),$$

*where function $K_{d}\left(n\right)$ is given by Equation (35), and vector $n_{H}\left(t\right)\in R^{d^{2}-1}$ is the solution of the Cauchy problem*

(42) $$\frac{dn_{H}\left(t,t_{0}\right)}{dt}=b_{H}\left(t\right)+n_{⊥}\left(t,t_{0}\right),\;\;\;n_{H}\left(t_{0},t_{0}\right)=0,$$

*with $n_{⊥}\left(t,t_{0}\right)\in R^{d^{2}-1}$ satisfying for all $t>t_{0}$ the orthogonality relation $n_{⊥}\left(t,t_{0}\right)\cdot \nabla_{n_{H}}(K_{d}\left(n_{H}\left(t,t_{0}\right)\right)=0$ and determined via the equation*

(43) $$\begin{array}{cc} \; & \frac{dn_{H}\left(t,t_{0}\right)}{dt}\cdot \frac{\partial }{\partial n_{H}}\left(\frac{\partial }{\partial n_{j}}K_{d}(n_{H}\left(t,t_{0}\right)\right)\\ \; & =-b_{H}^{\left(j\right)}\left(t\right)K_{d}\left(n_{H}\left(t,t_{0}\right)\right)\;+\;\sqrt{\frac{d}{2}}\sum_{k,m}\left(\;f_{kmj}-id_{kmj}\right)b_{H}^{\left(k\right)}\left(t\right)\frac{\partial }{\partial n_{H}^{\left(m\right)}}K_{d}\left(n_{H}\left(t,t_{0}\right)\right). \end{array}$$

In Section 4 and Section 5, we specify Equations (26), (42) and (43) for a general qubit case.

### Finding $K_{d}\left(n\right)$ for d=2,3

In this subsection, we consider the characteristic function $K_{d}\left(r\right)$, given by Equation (35), and also, representation (29) for d=2,3.

- For d=2, the matrix representations of generators $\sigma_{1},\sigma_{2},\sigma_{3}$ of SU(2) in the computational basis in $C^{2}$ are given by the Pauli matrices
  (44) $$\begin{array}{cc} \sigma_{1} & =\left(\begin{array}{cc} 0 & 1\\ 1 & 0 \end{array}\right),\;\;\;\;\sigma_{2}=\left(\begin{array}{cc} 0 & -i\\ i & 0 \end{array}\right),\;\;\;\sigma_{3}=\left(\begin{array}{cc} 1 & 0\\ 0 & -1 \end{array}\right),\\ \sigma & =\left(\sigma_{1},\sigma_{2},\sigma_{3}\right),\;\;\;\mathrm{tr}\left[\sigma_{k}\sigma_{j}\right]=2\delta_{jk},\\ \sigma_{1}\sigma_{2} & =i\sigma_{3},\;\;\;\sigma_{2}\sigma_{3}=i\sigma_{1},\;\;\;\sigma_{3}\sigma_{1}=i\sigma_{2}, \end{array}$$
  and, for each vector $r\in R^{3},$ the traceless Hermitian operator n·σ on $C^{2}$ has eigenvalues $\pm {∥n∥}_{R^{3}}.$ Therefore, by Equation (35), the characteristic function $K_{2}\left(r\right)$ and its derivatives are given by
  (45) $$\begin{array}{cc} K_{2}\left(r\right) & =\exp\left\{-i\;{∥r∥}_{R^{3}}\right\}+\exp\left\{i\;{∥r∥}_{R^{3}}\right\}\\ \; & =2\cos{∥r∥}_{R^{3}},\\ \frac{\partial }{\partial r_{j}}K_{2}\left(r\right) & =-2\sin\left({∥r∥}_{R^{3}}\right)\frac{r_{j}}{{∥r∥}_{R^{3}}}, \end{array}$$
  and representation (29) reduces to the well-known formula
  (46) $$\exp\left\{-i\;\left(r\cdot \sigma \right)\right\}=I\cos{∥r∥}_{R^{3}}-i\sin\left({∥r∥}_{R^{3}}\right)\frac{r\cdot \sigma }{{∥r∥}_{R^{3}}}$$
  (see, for example, Reference [13]).
- For d=3, the matrix representations of the SU(3) generators in the computational basis in $C^{3}$ constitute the Gell-Mann matrices. For each $r\in R^{8},$ the traceless Hermitian operator $\left(r\cdot \Lambda \right)$ on $C^{3}$ has eigenvalues [12]
  (47) $$\begin{array}{cc} \lambda_{1,2}\left(r\right) & =\frac{2}{\sqrt{3}}{∥r∥}_{R^{8}}\sin\left(\phi \left(r\right)\pm \frac{\pi }{3}\right),\\ \lambda_{3}\left(r\right) & =-\frac{2}{\sqrt{3}}{∥r∥}_{R^{8}}\sin\left(\phi (r)\right), \end{array}$$
  where
  (48) $$\sin\left(3\phi (r)\right)=-\frac{3\sqrt{3}}{2{∥r∥}_{R^{8}}^{3}}\det\left(r\cdot \Lambda \right).$$
  From relations (35) and (47), it follows that, for d=3,
  (49) $$\begin{array}{cc} K_{3}\left(r\right) & =\exp\left\{-i\sqrt{\frac{3}{2}}\lambda_{1}\left(r\right)\right\}+\exp\left\{-i\sqrt{\frac{3}{2}}\;\lambda_{2}\left(r\right)\right\}+\exp\left\{-i\;\sqrt{\frac{3}{2}}\lambda_{3}\left(r\right)\right\}\\ \; & =\sum_{k=0,1,2}\exp\left\{-i\sqrt{2}{∥r∥}_{R^{8}}\sin\left(\phi \left(r\right)+2\pi k/3\right)\right\} \end{array}$$
  and (see Appendix B)
  (50) $$\frac{\partial }{\partial r}K_{3}\left(r\right)=-3i\sqrt{\frac{2}{3}}\left(F_{1}\left(r\right)\;p\left(r\right)+F_{2}\left(r\right)\frac{\;r}{{∥r∥}_{R^{8}}}\right),$$
  where
  (51) $$\begin{array}{cc} p^{\left(m\right)}\left(r\right) & :=\sum_{i,j=1}^{8}\frac{r^{\left(i\right)}r^{\left(j\right)}d_{ijm}}{{∥r∥}_{R^{8}}^{2}},\;\;\;p^{\prime }\left(r\right)\in C^{8},\\ F_{1}\left(r\right) & :=\sum_{k=0,1,2}\frac{\exp\left\{-i\sqrt{2}{∥r∥}_{R^{8}}\sin\left(\phi \left(r\right)+2\pi k/3\right)\right\}}{1-2\cos(2(\phi (r)+2\pi k/3))},\\ F_{2}\left(r\right) & :=\frac{2}{\sqrt{3}}\sum_{k=0,1,2}\sin\left(\phi (r)+2\pi k/3\right)\\ \; & \times \frac{\exp\left\{-i\sqrt{2}{∥r∥}_{R^{8}}\sin\left(\phi \left(r\right)+2\pi k/3\right)\right\}}{1-2\cos(\left(2(\phi (r)+2\pi k/3)\right)}. \end{array}$$
  Taking into account Equations (37), (49) and (50), we derive that, for any vector $r\in R^{8},$
  (52) $$\exp\left\{-i\sqrt{\frac{3}{2}}\;\left(r\cdot \Lambda \right)\right\}=\frac{I}{3}K_{3}\left(r\right)+\left\{\;F_{1}\left(\phi (r)\right)\;\left(p(r)\cdot \Lambda \right)+F_{2}\left(\phi (r)\right)\;\left(r\cdot \Lambda \right)\;\right\}$$
  In view of relations (10) and (51), this expression can be otherwise represented in the form
  (53) $$\begin{array}{cc} \exp\left\{-i\sqrt{\frac{3}{2}}\;\left(r\cdot \Lambda \right)\right\} & =\sum_{k=0,1,2}\{\frac{1}{{∥r∥}_{R^{8}}^{2}}{\left(r\cdot \Lambda \right)}^{2}+\frac{2}{\sqrt{3}{∥r∥}_{R^{8}}}\left(r\cdot \Lambda \right)\sin\left(\phi (r)+2\pi k/3\right)\\ \; & -\frac{I}{3}\left[1+2\cos\left(2(\phi (r)+2\pi k/3)\right)\right]\}\\ \; & \times \frac{\exp\left\{-i\sqrt{2}{∥r∥}_{R^{8}}\sin\left(\phi (r)+2\pi k/3\right)\right\}}{1-2\cos\left(2(\phi (r)+2\pi k/3)\right)}, \end{array}$$
  which agrees with formula (5) in Reference [14].

## 4. General Nonstationary Qubit Case

In this section, based on the new general results derived in Section 2 and Section 3, we specify the unitary evolution operator (Equation (40)) for d=2.

In the qubit case, $\Lambda \equiv \sigma =(\sigma_{1},\sigma_{2},\sigma_{3})$ and a general Hamiltonian on $C^{2}$ has the form

(54) $$H\left(t\right)=b_{0}\left(t\right)I+b\left(t\right)\cdot \sigma ,\;\;\;\;b\left(t\right)\in R^{3}.$$

Here and in what follows, in short, we suppress the lower index *H* in notations $n_{H}\left(t\right)$, $b_{H}\left(t\right)\in R^{3}$ and the lower index $R^{3}$ in notation ${∥\cdot ∥}_{R^{3}}.$

Let us specify the main issues of Theorem 1 if d=2. In this case:

- The structure coefficients $d_{kmj}=0$, for all k,m,j=1,2,3, and coefficients $f_{kmj}=\epsilon_{kmj}$ constitute the Levi-Civita symbol. Therefore, the system of linear ODEs (Equation (26)) reduces to
  (55) $$\begin{array}{cc} {\overset{\cdot }{u}}_{0}\left(t,t_{0}\right) & =b\left(t\right)\cdot \tilde{u}\left(t,t_{0}\right),\;\;\;\;{\tilde{u}}_{0}\left(t_{0},t_{0}\right)=1,\\ \overset{\cdot }{\tilde{u}}\left(t,t_{0}\right) & =-u_{0}\left(t,t_{0}\right)b_{j}+b\left(t\right)\times \tilde{u}\left(t,t_{0}\right),\;\;\;\;\tilde{u}\left(t_{0},t_{0}\right)=0,\\ {\left(u_{0}\left(t,t_{0}\right)\right)}^{2}+{∥\tilde{u}\left(t,t_{0}\right)∥}_{R^{3}}^{2} & =1, \end{array}$$
  with $u_{0}\left(t,t_{0}\right)\in R$, $\tilde{u}\left(t,t_{0}\right)\in R^{3}$ and notation $b\times \tilde{u}\;$ for a vector product on $R^{3}$.
  By introducing a 4-dimensional real-valued unit vector $q\left(t,t_{0}\right)=\left(u_{0}\left(t,t_{0}\right),\tilde{u}\left(t,t_{0}\right)\right)\in R^{4}$ and denoting by $q^{\prime }\left(t,t_{0}\right)$ the column-vector with elements comprised of components of vector $q(t,t_{0}),$ we rewrite the system of linear ODEs (Equation (55)) in the normal form
  (56) $$\frac{d}{dt}q^{\prime }\left(t,t_{0}\right)=A\left(t\right)q^{\prime }\left(t,t_{0}\right),\;\;\;q\left(t_{0},t_{0}\right)=\left(1,0,0,0\right),$$
  with the skew-symmetric matrix
  (57) $$A\left(t\right)=\left(\begin{array}{cccc} 0 & b_{1}\left(t\right) & b_{2}\left(t\right) & b_{3}\left(t\right)\\ -b_{1}\left(t\right) & 0 & -b_{3}\left(t\right) & b_{2}\left(t\right)\\ -b_{2}\left(t\right) & b_{3}\left(t\right) & 0 & -b_{1}\left(t\right)\\ -b_{3}\left(t\right) & -b_{2}\left(t\right) & b_{1}\left(t\right) & 0 \end{array}\right).$$
- For d=2, function (Equation (35)) and its gradient are given due to Equation (45) by $K_{2}\left(n\right)=\cos∥n(t)∥,$$\nabla_{n}K_{2}\left(n\right)=-2\sin\left(∥n∥\right)\frac{n}{∥n∥},$, so that by Equation (41),
  (58) $$u_{0}\left(t,t_{0}\right)=\cos∥n(t,t_{0})∥\in R,\;\;\;\tilde{u}\left(t,t_{0}\right)=-\sin\left(∥n(t,t_{0})∥\right)\frac{n(t,t_{0})}{∥n(t,t_{0})∥}\in R^{3},$$
  and the first and the second equations in Equation (55) take the forms
  (59) $$\frac{d∥n(t,t_{0})∥}{dt}=\frac{b\left(t\right)\cdot n\left(t,t_{0}\right)}{∥n(t)∥}$$
  and
  (60) $$\begin{array}{cc} \; & \left(\frac{\sin∥n(t,t_{0})∥}{∥n(t,t_{0})∥}-\cos∥n(t)∥\right)\frac{d∥n(t,t_{0})∥}{dt}\frac{n(t,t_{0})}{∥n(t,t_{0})∥}-\frac{\sin∥n(t,t_{0})∥}{∥n(t,t_{0})∥}\frac{dn(t,t_{0})}{dt}\\ \; & =-b\cos∥n(t,t_{0})∥\;+\;\frac{b\left(t\right)\times n\left(t,t_{0}\right)}{∥n(t,t_{0})∥}\sin∥n(t,t_{0})∥, \end{array}$$
  respectively.
- The Cauchy problem (Equation (42)) in Theorem 1 reduces to
  (61) $$\begin{array}{cc} \frac{dn(t,t_{0})}{dt} & =b\left(t\right)+n_{⊥}\left(t,t_{0}\right)\;\;\;t>t_{0},\\ n(t_{0},t_{0}) & =0, \end{array}$$
  where vector $n_{⊥}\left(t\right)\in R^{3}$ is orthogonal for all $t>t_{0}$ to vector $n\left(t\right)\in R^{3}$ and is determined via Equation (43). For d=2, the latter equation reduces to
  (62) $$\begin{array}{cc} \; & \left(\frac{\sin∥n(t,t_{0})∥}{∥n(t,t_{0})∥}-\cos∥n(t,t_{0})∥\right)\left(\frac{b\left(t\right)\cdot n\left(t,t_{0}\right)}{{∥n(t,t_{0})∥}^{2}}n\left(t,t_{0}\right)\;-\;b\left(t\right)\right)\\ \; & =\frac{\sin∥n(t,t_{0})∥}{∥n(t,t_{0})∥}n_{⊥}\left(t,t_{0}\right)\;+\;\left(b\left(t\right)\times n\left(t,t_{0}\right)\right)\frac{\sin∥n(t,t_{0})∥}{∥n(t,t_{0})∥}. \end{array}$$
  Noting that, on the left-hand side of Equation (62), where
  (63) $$\frac{b\cdot n}{{∥n∥}^{2}}n\;\;-\;b=-\frac{1}{{∥n∥}^{2}}\left(n\times b\times n\right),$$
  and vectors
  (64) n×b×n,b×n
  are mutually orthogonal and are both in the plane orthogonal to vector $n\left(t,t_{0}\right)\in R^{3},$ we represent vector $n_{⊥}\left(t,t_{0}\right)$ in Equations (61) and (62) as
  (65) $$n_{⊥}\left(t,t_{0}\right)=\alpha \left(t\right)\left(b\left(t\right)\times n\left(t,t_{0}\right)\right)\;+\;\beta \left(t,t_{0}\right)\left(\;n\left(t,t_{0}\right)\times b\left(t\right)\times n\left(t,t_{0}\right)\right)$$
  and find via Equation (62) that
  (66) $$\alpha \left(t,t_{0}\right)=-1,\;\;\;\;\;\;\beta \left(t,t_{0}\right)=-\frac{1-∥n(t,t_{0})∥\mathrm{ctg}∥n(t,t_{0})∥}{{∥n(t,t_{0})∥}^{2}}.$$
  Therefore, Equation (61)–(66) imply
  (67) $$\begin{array}{cc} \frac{dn(t,t_{0})}{dt} & =b\left(t\right)-\left(b\left(t\right)\times n\left(t,t_{0}\right)\right)\\ \; & -\;\frac{1-∥n(t,t_{0})∥\mathrm{ctg}∥n(t,t_{0})∥}{{∥n(t,t_{0})∥}^{2}}\left(n\left(t,t_{0}\right)\times b\left(t\right)\times n\left(t,t_{0}\right)\right),\\ n(t_{0},t_{0}) & =0. \end{array}$$

Theorem 1 and relations in Equations (55)–(67) prove the following statement on the unitary evolution of a qubit in a general nonstationary case.

**Theorem** **2.** *Let $H\left(t\right)=b_{0}\left(t\right)I+b\left(t\right)\cdot \sigma ,$$b\left(t\right)\in R^{3},$ be a qubit Hamiltonian on $C^{2}$. The unitary operator $U_{H}\left(t,t_{0}\right)$ on $C^{2}$ describing the evolution of a qubit under Hamiltonian H(t) takes the form*

(68) $$\begin{array}{cc} U_{H}\left(t,t_{0}\right) & =\exp\left\{-i\int_{t_{0}}^{t}b_{0}\left(\tau \right)d\tau \right\}\exp\left\{-i\;\left(n(t,t_{0})\cdot \sigma \right)\right\}\\ \; & =\exp\left\{-i\int_{t_{0}}^{t}b_{0}\left(\tau \right)d\tau \right\}\left(\;u_{0}\left(t,t_{0}\right)\;I\;+\;i\;\tilde{u}\left(t,t_{0}\right)\cdot \sigma \right), \end{array}$$

*where the unit vector $\left(u_{0}\left(t,t_{0}\right),\tilde{u}\left(t,t_{0}\right)\right)\in R^{4}$ is the solution of the Cauchy problem (Equation (55)) (equivalently, Equation (56)), vector $n\left(t,t_{0}\right)\in R^{3}$ is the solution of the Cauchy problem (Equation (67)), and the following relations hold*

(69) $$\begin{array}{cc} u_{0}\left(t,t_{0}\right) & =\cos∥n(t,t_{0})∥\in R,\;\;\;\tilde{u}\left(t,t_{0}\right)=-\sin\left(∥n(t,t_{0})∥\right)\frac{n(t,t_{0})}{∥n(t,t_{0})∥},\\ \frac{n(t,t_{0})}{∥n(t,t_{0})∥} & =-\frac{\;\tilde{u}\left(t,t_{0}\right)}{\;∥\tilde{u}\left(t,t_{0}\right)∥},\;\;\;∥n(t,t_{0})∥=\arccos\left(u_{0}\left(t,t_{0}\right)\right). \end{array}$$

The cocycle property (Equation (3)) implies that, in the qubit case, the unit vector $\left(u_{0}\left(t,t_{0}\right),\tilde{u}\left(t,t_{0}\right)\right)\in R^{4}$ in Equation (68)—which is the solution of the Cauchy problem (56)—must satisfy the relations

(70) $$\begin{array}{cc} u_{0}\left(t,s\right)u_{0}\left(s,t_{0}\right)\;-\;\tilde{u}\left(t,s\right)\cdot \tilde{u}\left(s,t_{0}\right) & =u_{0}\left(t,t_{0}\right),\\ u_{0}\left(t,s\right)\tilde{u}\left(s,t_{0}\right)+u_{0}\left(s,t_{0}\right)\tilde{u}\left(t,s\right)\;-\;\tilde{u}\left(t,s\right)\times \tilde{u}\left(s,t_{0}\right) & =\tilde{u}\left(t,t_{0}\right). \end{array}$$

For d=2, relations in Equation (14) reduce to the condition

(71) $$b\left(t\right)\times \left(\int_{t_{0}}^{t}b\left(\tau \right)d\tau \right)=0,$$

which is necessary and sufficient for the Cauchy problem (Equation (55); equivalently, Equation (56)) and the Cauchy problem (in Equation (67)) to have the solutions

(72) $$\begin{array}{cc} n(t,t_{0}) & =\int_{t_{0}}^{t}b\left(\tau \right)d\tau ,\;\;\;\;\;u_{0}\left(t,t_{0}\right)=\cos\left(∥\int_{t_{0}}^{t}b\left(\tau \right)d\tau ∥\right),\\ \tilde{u}\left(t,t_{0}\right) & =-\frac{\sin\left(∥\int_{t_{0}}^{t}b\left(\tau \right)d\tau ∥\right)}{∥\int_{t_{0}}^{t}b\left(\tau \right)d\tau ∥}\left(\int_{t_{0}}^{t}b\left(\tau \right)d\tau \right), \end{array}$$

and the unitary evolution operator $U_{H}\left(t,t_{0}\right)$ to be given by

(73) $$\begin{array}{cc} U_{H}\left(t,t_{0}\right) & =\exp\left\{-i\int_{t_{0}}^{t}b_{0}\left(\tau \right)d\tau \right\}\exp\left\{-i\;\left(\int_{t_{0}}^{t}\left(b(\tau )\cdot \sigma \right)d\tau \right)\right\}\\ & =\exp\left\{-i\int_{t_{0}}^{t}b_{0}\left(\tau \right)d\tau \right\}\left[I\cos\left(∥\int_{t_{0}}^{t}b\left(\tau \right)d\tau ∥\right)-\;i\frac{\sin\left(∥\int_{t_{0}}^{t}b\left(\tau \right)d\tau ∥\right)}{∥\int_{t_{0}}^{t}b\left(\tau \right)d\tau ∥}\left(\int_{t_{0}}^{t}b\left(\tau \right)d\tau \cdot \sigma \right)\right]. \end{array}$$

The expression standing in the first line of Equation (73) is consistent with expression (15) valid under the general qudit condition (6) and specified for d=2.

Condition (71) is, in particular, true if $b\left(t\right)=e_{b}∥b(t)∥$ where a unit vector $e_{b}$ does not vary in time. Substituting this b(t) into Equation (73), we have

(74) $$\begin{array}{cc} U_{H}\left(t,t_{0}\right) & =\exp\left\{-i\int_{t_{0}}^{t}b_{0}\left(\tau \right)d\tau \right\}\exp\left\{-i\;\left(e_{b}\cdot \sigma \right)\int_{t_{0}}^{t}∥b(\tau )∥d\tau \right\}\\ \; & =\exp\left\{-i\int_{t_{0}}^{t}b_{0}\left(\tau \right)d\tau \right\}\left[I\cos\left(\int_{t_{0}}^{t}∥b(\tau )∥d\tau \right)\;-\;i\sin\left(\int_{t_{0}}^{t}∥b(\tau )∥d\tau \right)\left(e_{b}\cdot \sigma \right)\right]. \end{array}$$

In the following section, based on the general result formulated in Theorem 2, we specify classes of nonstationary Hamiltonians H(t), for which we can find the precise solutions of the Cauchy problem (in Equation (55); equivalently, (56)) and, hence, explicitly specify the unitary operator (68) via coefficients $b_{0}\left(t\right),$b(t) of a Hamiltonian H(t).

## 5. Special Classes of Qubit Hamiltonians

Let, for a qubit Hamiltonian (54), components $\left(∥b(t)∥,\theta_{b}\left(t\right),\varphi_{b}\left(t\right)\right)$ of a vector $b\left(t\right)\in R^{3}$ in the spherical coordinate system be such that (here, we suppose that b(t) is twice differentiable)

(75) $$\begin{array}{cc} \frac{d}{dt}J_{1} & =0,\;\;\;\mathrm{where}\;J_{1}:=\frac{1}{\Omega_{b}\left(t\right)}\left(\cos\left(\theta_{b}\left(t\right)\right)-\frac{{\overset{\cdot }{\varphi }}_{b}\left(t\right)}{2∥b(t)∥}\right),\\ \frac{d}{dt}J_{2} & =0,\;\;\;\mathrm{where}\;J_{2}:=\frac{\sin\left(\theta_{b}\left(t\right)\right)}{\Omega_{b}\left(t\right)}, \end{array}$$

where

(76) $$\Omega_{b}\left(t\right):=\sqrt{{\left(\cos\left(\theta_{b}\left(t\right)\right)-\frac{\overset{\cdot }{\varphi_{b}}\left(t\right)}{2∥b(t)∥}\right)}^{2}+\sin^{2}\left(\theta_{b}\left(t\right)\right)},$$

so that $J_{1}^{2}+J_{2}^{2}=1.$

The class of Hamiltonians specified by conditions (75) is rather broad and includes, in particular, all cases studied in the literature for which:

(77) $${\overset{\cdot }{\theta }}_{b}\left(t\right)=0,\;\;\;\overset{\cdot \cdot }{\varphi_{b}}\left(t\right)=0.$$

Represented otherwise, constant $J_{1}$ takes the form

(78) $$\begin{array}{cc} J_{1} & =\frac{1}{∥b(t)∥\Omega_{b}\left(t\right)}\left(b_{3}\left(t\right)-\frac{1}{2}\frac{d}{dt}\varphi_{b}\left(t\right)\right),\\ ∥b(t)∥\Omega_{b}\left(t\right) & =\sqrt{{\left(b_{3}\left(t\right)-\;\frac{1}{2}\frac{d}{dt}\varphi_{b}\left(t\right)\right)}^{2}+b_{1}^{2}\left(t\right)+b_{2}^{2}\left(t\right)},\\ \mathrm{tg}(\varphi_{b}\left(t\right)) & =b_{2}\left(t\right)/b_{1}\left(t\right), \end{array}$$

from which it is immediately clear that the class of Hamiltonians specified by conditions (75) is defined via the special time behavior of a vector b(t) with respect to the $x_{3}$-axis.

Quite similarly, we can introduce the class of Hamiltonians specified via the property of $b\left(t\right)\in R^{3}$ which is similar by its form to (78) but with respect to the x${}_{1}$-axis or the x${}_{2}$-axis.

Though, in the following statement, we explicitly specify only the unitary qubit evolution (68) under a Hamiltonian satisfying conditions (75), the new result of this statement can be easily reformulated for the classes of nonstationary Hamiltonians specified by conditions on $b\left(t\right)\in R^{3}$ with respect to the x${}_{1}$-axis and the x${}_{2}$-axis.

**Theorem** **3.** *Let, for a qubit Hamiltonian $H\left(t\right)=b_{0}\left(t\right)I+b\left(t\right)\cdot \sigma$ on $C^{2}$ the conditions (75) be fulfilled. Then, for the unitary operator $U_{H}\left(t,t_{0}\right)$ given by relations (68) and (69) and describing the evolution of a qubit state under a Hamiltonian H(t), the unit vector $\left(u_{0}\left(t,t_{0}\right),\tilde{u}\left(t,t_{0}\right)\right)\in R^{4}$—the solution of the Cauchy problem (55), equivalently, Equation (56), takes the form*

(79) $$\begin{array}{cc} u_{0}\left(t,t_{0}\right) & =\cos\left(\frac{\varphi_{b}\left(t\right)-\varphi_{b}\left(t_{0}\right)}{2}\right)\cos\left(\gamma_{b}\left(t,t_{0}\right)\right)-J_{1}\sin\left(\frac{\varphi_{b}\left(t\right)-\varphi_{b}\left(t_{0}\right)}{2}\right)\sin\left(\gamma_{b}\left(t,t_{0}\right)\right),\\ {\tilde{u}}_{1}\left(t,t_{0}\right) & =-J_{2}\cos\left(\frac{\varphi_{b}\left(t\right)+\varphi_{b}\left(t_{0}\right)}{2}\right)\sin\left(\gamma_{b}\left(t,t_{0}\right)\right),\\ {\tilde{u}}_{2}\left(t,t_{0}\right) & =-J_{2}\sin\left(\frac{\varphi_{b}\left(t\right)+\varphi_{b}\left(t_{0}\right)}{2}\right)\sin\left(\gamma_{b}\left(t,t_{0}\right)\right),\\ {\tilde{u}}_{3}\left(t,t_{0}\right) & =-J_{1}\cos\left(\frac{\varphi_{b}\left(t\right)-\varphi_{b}\left(t_{0}\right)}{2}\right)\sin\left(\gamma_{b}\left(t,t_{0}\right)\right)-\sin\left(\frac{\varphi_{b}\left(t\right)-\varphi_{b}\left(t_{0}\right)}{2}\right)\cos\left(\gamma_{b}\left(t,t_{0}\right)\right), \end{array}$$

*satisfying the cocycle property (70). In Equation (79),*

(80) $$\gamma_{b}\left(t,t_{0}\right):=\int_{t_{0}}^{t}∥b(\tau )∥\Omega_{b}\left(\tau \right)\;d\tau$$

*and $\theta_{b}\left(t\right),$$\varphi_{b}\left(t\right)$ are angles specifying at time t vector $b\left(t\right)\in R^{3}$ in the spherical coordinate system.*

The proof of this statement is given in Appendix A. Note that the Cauchy problem with a skew-symmetric matrix—like the one in Equation (56)—arises in many fields of mathematical physics, for example, in the solid body theory, in the quaternions models [15], etc. If we reformulate conditions (78) (equivalently, Equation (75)) with respect to the x${}_{1}$-axis, then the corresponding solution $\left(u_{0}\left(t,t_{0}\right),\tilde{u}\left(t,t_{0}\right)\right)\in R^{4}$ of the Cauchy problem for the ODEs (56) would agree with the treatment in Section 5.10 of Ref. [15].

Let, for example, $b\left(t\right)=e_{b}∥b(t)∥$ where a unit vector $e_{b}$ does not vary in time—the case we have analyzed above in Equation (74) and where the general condition (71) is true. In this case,

(81) $$\begin{array}{cc} \varphi_{b}\left(t\right) & =\varphi_{b}\left(t_{0}\right)=\varphi_{b},\;\;\;\;\theta_{b}\left(t\right)=\theta_{b}\left(t_{0}\right)=\theta_{b},\\ J_{1} & =\cos\theta_{b},\;\;\;J_{2}=\sin\theta_{b}, \end{array}$$

conditions (75) are also fulfilled, and the substitution of Equation (81) into expression (78) leads exactly to relation (73).

However, in general, conditions (71) and (75) do not need to be fulfilled simultaneously.

As an application of the result of Theorem 3, consider some examples important for applications where conditions (75) are fulfilled while condition (71) is violated.

1. Let, for a qubit Hamiltonian, as in Equation (54), the spherical coordinates of a vector $b\left(t\right)\in R^{3}$ satisfy the relations
  (82) $$\theta_{b}\left(t\right)=\theta_{b},\;\;\;\varphi_{b}\left(t\right)=\omega t+\eta ,\;\;\;∥b(t)∥=b,\;\;\;\eta \in R,$$
  in the case where a vector b(t) rotates around the $x_{3}$-axis with an angular velocity ω and has a norm constant in time. Based on approaches different to ours, this case was considered in many papers in connection with the evolution of a pure qubit state; see, for example, Reference [3]. For case (82), conditions (75) and parameters in (79) take the forms:
  (83) $$\begin{array}{cc} J_{1} & =\frac{\cos\theta_{b}-\omega /2b}{\Omega_{b}},\;\;\;\;J_{2}=\frac{\sin\theta_{b}}{\Omega_{b}},\\ \Omega_{b} & =\sqrt{{\left(\cos\theta_{b}-\omega /2b\right)}^{2}+\sin^{2}\theta_{b}}=Const,\\ ∥b(t)∥\Omega_{b} & =\sqrt{{\left(2b\cos\theta_{b}-\omega \right)}^{2}+4b^{2}\sin^{2}\theta_{b}}:={\tilde{\Omega }}_{b}. \end{array}$$
  Therefore, for case (82), we have by Theorem 3:
  (84) $$\begin{array}{cc} u_{0}\left(t,t_{0}\right) & =\cos\left(\frac{\omega (t-t_{0})}{2}\right)\cos\left({\tilde{\Omega }}_{b}\left(t-t_{0}\right)\right)-J_{1}\sin\left(\frac{\omega (t-t_{0})}{2}\right)\sin\left({\tilde{\Omega }}_{b}\left(t-t_{0}\right)\right),\\ {\tilde{u}}_{1}\left(t,t_{0}\right) & =-J_{2}\cos\left(\frac{\omega (t+t_{0})}{2}+\eta \right)\sin\left({\tilde{\Omega }}_{b}\left(t-t_{0}\right)\right),\\ {\tilde{u}}_{2}\left(t,t_{0}\right) & =-J_{2}\sin\left(\frac{\omega (t+t_{0})}{2}+\eta \right)\sin\left({\tilde{\Omega }}_{b}\left(t-t_{0}\right)\right),\\ {\tilde{u}}_{3}\left(t,t_{0}\right) & =-J_{1}\cos\left(\frac{\omega (t-t_{0})}{2}\right)\sin\left({\tilde{\Omega }}_{b}\left(t-t_{0}\right)\right)-\sin\left(\frac{\omega (t-t_{0})}{2}\right)\cos\left({\tilde{\Omega }}_{b}\left(t-t_{0}\right)\right), \end{array}$$
  so that the unitary evolution operator (68) with the unit vector $\left(u_{0}\left(t\right),\tilde{u}\left(t\right)\right)$ given by Equation (84) completely defines the evolution of every qubit state under a nonstanionary Hamiltonian specified by relations (82).
  Taking, for example, $t_{0}=0$ and an initial pure state $|\Psi \left(0\right)\rangle =|0\rangle \in C^{2}$, we find that at any moment t>0 the pure state is
  (85) $$\begin{array}{cc} |\Psi (t)\rangle & =U_{H}\left(t\right)|0\rangle =u_{0}\left(t,0\right)|0\rangle +i{\tilde{u}}_{1}\left(t,0\right)|1\rangle -{\tilde{u}}_{2}\left(t,0\right)|1\rangle +i{\tilde{u}}_{3}\left(t,0\right)|0\rangle \\ \; & =\left(u_{0}\left(t,0\right)+i{\tilde{u}}_{3}\left(t,0\right)\right)|0\rangle +i\left({\tilde{u}}_{1}\left(t,0\right)+i{\tilde{u}}_{2}\left(t,0\right)\right)|1\rangle , \end{array}$$
  where |0〉,|1〉 are elements of the computational basis of $C^{2}.$ Substituting (84) into Equation (85), we have
  (86) $$\begin{array}{cc} u_{0}\left(t,0\right)+i{\tilde{u}}_{3}\left(t,0\right) & =\left(\cos\left({\tilde{\Omega }}_{b}t\right)-iJ_{1}\sin\left({\tilde{\Omega }}_{b}t\right)\right)\exp\left\{-\frac{i\omega t}{2}\right\},\\ {\tilde{u}}_{1}\left(t,0\right)+i{\tilde{u}}_{2}\left(t,0\right) & =-J_{2}\sin\left({\tilde{\Omega }}_{b}t\right)\exp\left\{\frac{i\omega t}{2}+\eta \right\}, \end{array}$$
  so that
  (87) $$|\Psi \left(t\right)\rangle =\left\{\cos\left({\tilde{\Omega }}_{b}t\right)-iJ_{1}\sin\left({\tilde{\Omega }}_{b}t\right)\right\}\exp\left\{-\frac{i\omega t}{2}\right\}|0\rangle -iJ_{2}\sin\left({\tilde{\Omega }}_{b}t\right)\exp\left\{\frac{i\omega t}{2}+\eta \right\}|1\rangle ,$$
  where constants $J_{1}$ and $J_{2}$ are given by Equation (83). For η=0, the pure state (86) coincides with the pure state given by Equation (138.11) in Ref. [3] and found by another approach.
2. Consider further a more general case, where, for a vector $b\left(t\right)\in R^{3}$ in Equation (54):
  (88) $$b_{1}\left(t\right)=q\frac{{\overset{\cdot }{\varphi }}_{b}\left(t\right)}{\lambda }\cos\left(\varphi_{b}\left(t\right)\right),\;b_{2}\left(t\right)=q\frac{{\overset{\cdot }{\varphi }}_{b}\left(t\right)}{\lambda }\sin\left(\varphi_{b}\left(t\right)\right),\;b_{3}\left(t\right)=p\frac{{\overset{\cdot }{\varphi }}_{b}\left(t\right)}{\lambda },$$
  with function $\frac{{\overset{\cdot }{\varphi }}_{b}\left(t\right)}{\lambda }>0$ for all $t>t_{0}$ and some constants λ, q, *p*. In this case,
  (89) $$\begin{array}{cc} ∥b(t)∥ & =\frac{{\overset{\cdot }{\varphi }}_{b}\left(t\right)}{\lambda }\sqrt{q^{2}+p^{2}},\;\;\;\cos\left(\theta_{b}\left(t\right)\right)=\frac{p}{\sqrt{q^{2}+p^{2}}}=Const,\\ \Omega_{b}\left(t\right) & =\frac{1}{\sqrt{q^{2}+p^{2}}}\sqrt{{\left(p-\frac{\lambda }{2}\right)}^{2}+q^{2}}=Const,\\ ∥b(t)∥\Omega_{b} & =\frac{{\overset{\cdot }{\varphi }}_{b}\left(t\right)}{\lambda }\sqrt{{\left(p-\frac{\lambda }{2}\right)}^{2}+q^{2}}=\frac{\zeta }{\lambda }{\overset{\cdot }{\varphi }}_{b}\left(t\right),\\ \zeta & :=\sqrt{{\left(p-\frac{\lambda }{2}\right)}^{2}+q^{2}}=Const. \end{array}$$
  Hence, by Equation (75) the constants
  (90) $$J_{1}=\frac{p-\lambda /2}{\zeta },\;\;\;\;J_{2}=\frac{q}{\zeta },$$
  and, in Theorem 3, the vector $\left(u_{0}\left(t\right),\tilde{u}\left(t\right)\right)\in R^{4},$ which specifies by Equation (68) the unitary evolution of a qubit, is given by
  (91) $$\begin{array}{cc} u_{0}\left(t,t_{0}\right) & =\cos\left(\frac{\varphi_{b}\left(t\right)-\varphi_{b}\left(t_{0}\right)}{2}\right)\cos\left\{\frac{\zeta }{\lambda }\left(\varphi_{b}\left(t\right)-\varphi_{b}\left(t_{0}\right)\right)\right\}\\ \; & -\frac{p-\lambda /2}{\zeta }\sin\left(\frac{\varphi_{b}\left(t\right)-\varphi_{b}\left(t_{0}\right)}{2}\right)\sin\left\{\frac{\zeta }{\lambda }\left(\varphi_{b}\left(t\right)-\varphi_{b}\left(t_{0}\right)\right)\right\},\\ {\tilde{u}}_{1}\left(t,t_{0}\right) & =-\frac{q}{\zeta }\cos\left(\frac{\varphi_{b}\left(t\right)+\varphi_{b}\left(t_{0}\right)}{2}\right)\sin\left\{\frac{\zeta }{\lambda }(\varphi_{b}\left(t\right)-\varphi_{b}\left(t_{0}\right)\right\},\\ {\tilde{u}}_{2}\left(t,t_{0}\right) & =-\frac{q}{\zeta }\sin\left(\frac{\varphi_{b}\left(t\right)+\varphi_{b}\left(t_{0}\right)}{2}\right)\sin\left\{\frac{\zeta }{\lambda }\left(\varphi_{b}\left(t\right)-\varphi_{b}\left(t_{0}\right)\right)\right\},\\ {\tilde{u}}_{3}\left(t,t_{0}\right) & =-\frac{p-\lambda /2}{\zeta }\cos\left(\frac{\varphi_{b}\left(t\right)-\varphi_{b}\left(t_{0}\right)}{2}\right)\sin\left\{\frac{\zeta }{\lambda }\left(\varphi_{b}\left(t\right)-\varphi_{b}\left(t_{0}\right)\right)\right\}\\ \; & +\sin\left(\frac{\varphi_{b}\left(t\right)-\varphi_{b}\left(t_{0}\right)}{2}\right)\cos\left\{\frac{\zeta }{\lambda }\left(\varphi_{b}\left(t\right)-\varphi_{b}\left(t_{0}\right)\right)\right\}, \end{array}$$
  where λ,q,*p* are some constants and angle $\varphi_{b}\left(t\right)$ is an arbitrary function of *t*, such that $\frac{{\overset{\cdot }{\varphi }}_{b}\left(t\right)}{\lambda }>0$. If, in particular, ${\overset{\cdot }{\varphi }}_{b}\left(t\right)=\omega$ and λ=ω, then relations (91) reduce to relations (84).

## 6. Conclusions

In the present article, we introduced a new general formalism that allows for the analysis of the unitary evolution of a qudit (d≥2) under an arbitrary time-dependent Hamiltonian H(t) in terms of the Bloch-like vector space. Via this formalism, we derived (Theorem 1, Section 3) the new general equations specifying the evolution of the Bloch-like vector in the generalized Gell-Mann representation of $U_{H}\left(t,t_{0}\right)$ and the vector $n\left(t,t_{0}\right)\in R^{d^{2}-1}$ in the exponential representation of $U_{H}\left(t,t_{0}\right)$.

Applying the general Equations (26), (42), (43) to a qubit case (d=2), we then derived (Theorem 2, Section 4) a new general result on the qubit evolution under a nonstationary Hamiltonian. This general result allowed us to find (Theorem 3, Section 5) the new precise analytical solutions for a wide class of nonstationary Hamiltonians which comprise the qubit cases already known in the literature only as particular ones.

The general formalism presented in this article is valid for a qudit of an arbitrary dimension d>2, in particular, for a qutrit and the analysis of the evolution of a qutrit under a time-dependent Hamiltonian within this new formalism is a subject of our future research.

## Appendix A

In this section, we present the proof of Theorem 3, namely, we show that functions $u_{0}\left(t\right)\in R,$$\tilde{u}\left(t\right)\in R^{3},$ given by Equation (79), constitute solutions of the Cauchy problem (55), equivalently (56), under conditions (75) and satisfy also the cocycle property (70).

Under conditions (75), the derivative of function $u_{0}\left(t\right)\in R$ in Equation (79) has the form

(A1) $$\begin{array}{cc} \frac{d}{dt}u_{0}\left(t\right) & =-\frac{1}{2}\frac{d\varphi_{b}\left(t\right)}{dt}\sin\left(\frac{\varphi_{b}\left(t\right)-\varphi_{b}\left(t_{0}\right)}{2}\right)\cos\left(\gamma_{b}\left(t,t_{0}\right)\right)\\ \; & -∥b\left(t\right)∥\Omega_{b}\left(t\right)\cos\left(\frac{\varphi_{b}\left(t\right)-\varphi_{b}\left(t_{0}\right)}{2}\right)\sin\left(\gamma_{b}\left(t,t_{0}\right)\right)\\ \; & -\frac{J_{1}}{2}\frac{d\varphi_{b}\left(t\right)}{dt}\cos\left(\frac{\varphi_{b}\left(t\right)-\varphi_{b}\left(t_{0}\right)}{2}\right)\sin\left(\gamma_{b}\left(t,t_{0}\right)\right)\\ \; & -J_{1}∥b\left(t\right)∥\Omega_{b}\left(t\right)\sin\left(\frac{\varphi_{b}\left(t\right)-\varphi_{b}\left(t_{0}\right)}{2}\right)\cos\left(\gamma_{b}\left(t,t_{0}\right)\right)\\ \; & =-\left(J_{1}∥b\left(t\right)∥\Omega_{b}\left(t\right)+\frac{1}{2}\frac{d\varphi_{b}\left(t\right)}{dt}\right)\sin\left(\frac{\varphi_{b}\left(t\right)-\varphi_{b}\left(t_{0}\right)}{2}\right)\cos\left(\gamma_{b}\left(t,t_{0}\right)\right)\\ \; & -\left(∥b\left(t\right)∥\Omega_{b}\left(t\right)+\frac{J_{1}}{2}\frac{d\varphi_{b}\left(t\right)}{dt}\right)\cos\left(\frac{\varphi_{b}\left(t\right)-\varphi_{b}\left(t_{0}\right)}{2}\right)\sin\left(\gamma_{b}\left(t,t_{0}\right)\right). \end{array}$$

Similarly, for the derivatives of $\tilde{u}\left(t\right)=\left({\tilde{u}}_{1}\left(t\right),{\tilde{u}}_{2}\left(t\right),{\tilde{u}}_{3}\left(t\right)\right)\in R^{3},$ given by Equation (79), we find

(A2) $$\begin{array}{cc} \frac{d}{dt}{\tilde{u}}_{1}\left(t\right) & =-J_{2}∥b\left(t\right)∥\Omega_{b}\left(t\right)\cos\left(\frac{\varphi_{b}\left(t\right)+\varphi_{b}\left(t_{0}\right)}{2}\right)\cos\gamma_{b}\left(t,t_{0}\right)\\ \; & +\frac{J_{2}}{2}\frac{d\varphi_{b}\left(t\right)}{dt}\sin\left(\frac{\varphi \left(t\right)+\varphi \left(t_{0}\right)}{2}\right)\sin\gamma_{b}\left(t,t_{0}\right),\\ \frac{d}{dt}{\tilde{u}}_{2}\left(t\right) & =-J_{2}∥b\left(t\right)∥\Omega_{b}\left(t\right)\sin\left(\frac{\varphi_{b}\left(t\right)+\varphi_{b}\left(t_{0}\right)}{2}\right)\cos\gamma_{b}\left(t,t_{0}\right)\\ \; & -\frac{J_{2}}{2}\frac{d\varphi_{b}\left(t\right)}{dt}\cos\left(\frac{\varphi \left(t\right)+\varphi \left(t_{0}\right)}{2}\right)\sin\gamma_{b}\left(t,t_{0}\right),\\ \frac{d}{dt}{\tilde{u}}_{3}\left(t\right) & =-\left(J_{1}∥b\left(t\right)∥\Omega_{b}\left(t\right)+\frac{1}{2}\frac{d\varphi_{b}\left(t\right)}{dt}\right)\cos\left(\frac{\varphi_{b}\left(t\right)-\varphi_{b}\left(t_{0}\right)}{2}\right)\cos\gamma_{b}\left(t,t_{0}\right)\\ \; & +\left(∥b\left(t\right)∥\Omega_{b}\left(t\right)+\frac{J_{1}}{2}\frac{d\varphi_{b}\left(t\right)}{dt}\right)\sin\left(\frac{\varphi_{b}\left(t\right)-\varphi_{b}\left(t_{0}\right)}{2}\right)\sin\gamma_{b}\left(t,t_{0}\right). \end{array}$$

Next: (i) substituting Equation (79) into the terms standing on the right-hand sides of the equations in Equation (56); (ii) expressing $b_{1}\left(t\right),$$b_{2}\left(t\right),$ $b_{3}\left(t\right)$ in spherical coordinates; and (iii) using the trigonometric addition theorems and the explicit expressions for $J_{1}$, $J_{2}$ and $\Omega_{b}\left(t\right)$ (see Equation (75) and (76)), we derive the following expressions:

- for the right-hand side of the first differential equation in Equation (56)
  (A3) $$\begin{array}{cc} \; & b_{1}\left(t\right){\tilde{u}}_{1}\left(t\right)+b_{2}\left(t\right){\tilde{u}}_{2}\left(t\right)+b_{3}\left(t\right){\tilde{u}}_{3}\left(t\right)\\ \; & =-\left(J_{1}∥b\left(t\right)∥\Omega_{b}\left(t\right)+\frac{1}{2}\frac{d\varphi_{b}\left(t\right)}{dt}\right)\sin\left(\frac{\varphi_{b}\left(t\right)-\varphi_{b}\left(t_{0}\right)}{2}\right)\cos\left(\gamma_{b}\left(t,t_{0}\right)\right)\\ \; & -\left(∥b\left(t\right)∥\Omega_{b}\left(t\right)+\frac{J_{1}}{2}\frac{d\varphi_{b}\left(t\right)}{dt}\right)\cos\left(\frac{\varphi_{b}\left(t\right)-\varphi_{b}\left(t_{0}\right)}{2}\right)\sin\left(\gamma_{b}\left(t,t_{0}\right)\right); \end{array}$$
- for the right-hand side of the second differential equation in Equation (56)
  (A4) $$\begin{array}{cc} \; & -b_{1}\left(t\right)u_{0}\left(t\right)+b_{2}\left(t\right){\tilde{u}}_{3}\left(t\right)-b_{3}\left(t\right){\tilde{u}}_{2}\left(t\right)\\ \; & =-J_{2}∥b\left(t\right)∥\Omega_{b}\left(t\right)\cos\left(\frac{\varphi_{b}\left(t\right)+\varphi_{b}\left(t_{0}\right)}{2}\right)\cos\left(\gamma_{b}\left(t,t_{0}\right)\right)\\ \; & +\frac{J_{2}}{2}\frac{d\varphi_{b}\left(t\right)}{dt}\sin\left(\frac{\varphi \left(t\right)+\varphi \left(t_{0}\right)}{2}\right)\sin\left(\gamma_{b}\left(t,t_{0}\right)\right); \end{array}$$
- for the right-hand sides of the third and the fourth differential equations in Equation (56):
  (A5) $$\begin{array}{cc} \; & -b_{2}\left(t\right)u_{0}\left(t\right)+b_{3}\left(t\right){\tilde{u}}_{1}\left(t\right)-b_{1}\left(t\right){\tilde{u}}_{3}\left(t\right)\\ \; & =-J_{2}∥b\left(t\right)∥\Omega_{b}\left(t\right)\sin\left(\frac{\varphi_{b}\left(t\right)+\varphi_{b}\left(t_{0}\right)}{2}\right)\cos\left(\gamma_{b}\left(t,t_{0}\right)\right)\\ \; & -\frac{J_{2}}{2}\frac{d\varphi_{b}\left(t\right)}{dt}\cos\left(\frac{\varphi \left(t\right)+\varphi \left(t_{0}\right)}{2}\right)\sin\left(\gamma_{b}\left(t,t_{0}\right)\right) \end{array}$$
  and
  (A6) $$\begin{array}{cc} \; & -b_{3}\left(t\right)u_{0}\left(t\right)+b_{1}\left(t\right){\tilde{u}}_{2}\left(t\right)-b_{2}\left(t\right){\tilde{u}}_{1}\left(t\right)\\ \; & =-\left(J_{1}∥b\left(t\right)∥\Omega_{b}\left(t\right)+\frac{1}{2}\frac{d\varphi_{b}\left(t\right)}{dt}\right)\cos\left(\frac{\varphi_{b}\left(t\right)-\varphi_{b}\left(t_{0}\right)}{2}\right)\cos\left(\gamma_{b}\left(t,t_{0}\right)\right)\\ \; & +\left(∥b\left(t\right)∥\Omega_{b}\left(t\right)+\frac{J_{1}}{2}\frac{d\varphi_{b}\left(t\right)}{dt}\right)\sin\left(\frac{\varphi_{b}\left(t\right)-\varphi_{b}\left(t_{0}\right)}{2}\right)\sin\left(\gamma_{b}\left(t,t_{0}\right)\right). \end{array}$$

Clearly, the expressions for $\frac{d}{dt}u_{0}\left(t\right),$$\frac{d}{dt}{\tilde{u}}_{1}\left(t\right),$$\frac{d}{dt}{\tilde{u}}_{2}\left(t\right),$$\frac{d}{dt}{\tilde{u}}_{3}\left(t\right)$, derived in Equation (A1),(A2), coincide with the corresponding expressions in Equation (A3)–(A6). This proves that functions (79) constitute the solutions to the Cauchy problem (56), equivalently, Equation (55).

Taking into account that (see in Section 2) the unitary evolution operator $U_{H}\left(t,s\right)=u_{0}\left(t,s\right)I+i\tilde{u}\left(t,s\right)\cdot \sigma$, for each $s\in [t,t_{0}],$ let us now prove that solutions (79) satisfy the cocycle property (3) for $U_{H}\left(t,t_{0}\right).$ In terms of $u_{0}\left(t,s\right),$ $\tilde{u}\left(t,s\right),$ the cocycle property leads to relations (70), which read:

(A7) $$\begin{array}{cc} u_{0}\left(t,s\right)u_{0}\left(s,t_{0}\right)-\tilde{u}\left(t,s\right)\cdot \tilde{u}\left(s,t_{0}\right) & =u_{0}\left(t,t_{0}\right),\\ u_{0}\left(t,s\right)\tilde{u}\left(s,t_{0}\right)+u_{0}\left(s,t_{0}\right)\tilde{u}\left(t,s\right)-\tilde{u}\left(t,s\right)\times \tilde{u}\left(s,t_{0}\right) & =\tilde{u}\left(t,t_{0}\right). \end{array}$$

Substituting solutions (79) into Equation (A7), applying the addition rules for trigonometric functions, and taking into account that $J_{1}^{2}+J_{2}^{2}=1$, for the left-hand side of the first equation in Equation (A7), we derive:

(A8) $$\begin{array}{cc} \; & u_{0}\left(t,s\right)u_{0}\left(s,t_{0}\right)-\tilde{u}\left(t,s\right)\cdot \tilde{u}\left(s,t_{0}\right)\\ \; & =\cos\left(\gamma_{b}\left(t,s\right)\right)\cos\left(\gamma_{b}\left(s,t_{0}\right)\right)\cos\left(\frac{\varphi_{b}\left(t\right)-\varphi_{b}\left(t_{0}\right)}{2}\right)-J_{1}\sin\left(\frac{\varphi_{b}\left(t\right)-\varphi_{b}\left(t_{0}\right)}{2}\right)\\ \; & \times [\cos\left(\gamma_{b}\left(t,s\right)\right)\sin\left(\gamma_{b}\left(s,t_{0}\right)\right)+\sin\left(\gamma_{b}\left(t,s\right)\right)\cos\left(\gamma_{b}\left(s,t_{0}\right)\right)]\\ \; & -\sin\left(\gamma_{b}\left(t,s\right)\right)\sin\left(\gamma_{b}\left(s,t_{0}\right)\right)\cos\left(\frac{\varphi_{b}\left(t\right)-\varphi_{b}\left(t_{0}\right)}{2}\right) \end{array}$$

(A9) $$\begin{array}{cc} \; & =\cos\left(\frac{\varphi_{b}\left(t\right)-\varphi_{b}\left(t_{0}\right)}{2}\right)\cos\left(\gamma_{b}\left(t,t_{0}\right)\right)-J_{1}\sin\left(\frac{\varphi_{b}\left(t\right)-\varphi_{b}\left(t_{0}\right)}{2}\right)\sin\left(\gamma_{b}\left(t,t_{0}\right)\right). \end{array}$$

By the same procedure, for the left-hand sides of the remaining equations in Equation (A7), we have

(A10) $$\begin{array}{cc} \; & u_{0}\left(t,s\right){\tilde{u}}_{1}\left(s,t_{0}\right)+u_{0}\left(s,t_{0}\right){\tilde{u}}_{1}\left(t,s\right)-{\left(\tilde{u}\left(t,s\right)\times \tilde{u}\left(s,t_{0}\right)\right)}_{1}\\ \; & =-J_{2}[\sin\left(\gamma_{b}\left(t,s\right)\right)\cos\left(\gamma_{b}\left(s,t_{0}\right)\right)+\cos\left(\gamma_{b}\left(t,s\right)\right)\sin\left(\gamma_{b}\left(s,t_{0}\right)\right)]\cos\left(\frac{\varphi_{b}\left(t\right)+\varphi_{b}\left(t_{0}\right)}{2}\right)\\ \; & +J_{1}J_{2}\sin\left(\gamma_{b}\left(t,s\right)\right)\sin\left(\gamma_{b}\left(s,t_{0}\right)\right)[\sin\left(\frac{\varphi_{b}\left(t\right)-\varphi_{b}\left(s\right)}{2}\right)\cos\left(\frac{\varphi_{b}\left(s\right)+\varphi_{b}\left(t_{0}\right)}{2}\right)\\ \; & +\cos\left(\frac{\varphi_{b}\left(t\right)+\varphi_{b}\left(s\right)}{2}\right)\sin\left(\frac{\varphi_{b}\left(s\right)-\varphi_{b}\left(t_{0}\right)}{2}\right)+\cos\left(\frac{\varphi_{b}\left(t\right)-\varphi_{b}\left(s\right)}{2}\right)\\ \; & \times \sin\left(\frac{\varphi_{b}\left(s\right)+\varphi_{b}\left(t_{0}\right)}{2}\right)-\sin\left(\frac{\varphi_{b}\left(t\right)+\varphi_{b}\left(s\right)}{2}\right)\cos\left(\frac{\varphi_{b}\left(s\right)-\varphi_{b}\left(t_{0}\right)}{2}\right)] \end{array}$$

(A11) $$\begin{array}{ccc} \; & =-J_{2}\cos\left(\frac{\varphi_{b}\left(t\right)+\varphi_{b}\left(t_{0}\right)}{2}\right)\sin\left(\gamma_{b}\left(t,t_{0}\right)\right) & \; \end{array}$$

and

(A12) $$\begin{array}{cc} \; & u_{0}\left(t,s\right){\tilde{u}}_{2}\left(s,t_{0}\right)+u_{0}\left(s,t_{0}\right){\tilde{u}}_{2}\left(t,s\right)-{\left(\tilde{u}\left(t,s\right)\times \tilde{u}\left(s,t_{0}\right)\right)}_{2}\\ \; & =-J_{2}\left[\sin\left(\gamma_{b}\left(t,s\right)\right)\cos\left(\gamma_{b}\left(s,t_{0}\right)\right)+\cos\left(\gamma_{b}\left(t,s\right)\right)\sin\left(\gamma_{b}\left(s,t_{0}\right)\right)\right]\sin\left(\frac{\varphi_{b}\left(t\right)+\varphi_{b}\left(t_{0}\right)}{2}\right)\\ \; & +J_{1}J_{2}\sin\left(\gamma_{b}\left(t,s\right)\right)\sin\left(\gamma_{b}\left(s,t_{0}\right)\right)[\sin\left(\frac{\varphi_{b}\left(t\right)-\varphi_{b}\left(s\right)}{2}\right)\sin\left(\frac{\varphi_{b}\left(s\right)+\varphi_{b}\left(t_{0}\right)}{2}\right)\\ \; & +\sin\left(\frac{\varphi_{b}\left(t\right)+\varphi_{b}\left(s\right)}{2}\right)\sin\left(\frac{\varphi_{b}\left(s\right)-\varphi_{b}\left(t_{0}\right)}{2}\right)-\cos\left(\frac{\varphi_{b}\left(t\right)-\varphi_{b}\left(s\right)}{2}\right)\cos\left(\frac{\varphi_{b}\left(s\right)+\varphi_{b}\left(t_{0}\right)}{2}\right)\\ \; & +\cos\left(\frac{\varphi_{b}\left(t\right)+\varphi_{b}\left(s\right)}{2}\right)\cos\left(\frac{\varphi_{b}\left(s\right)-\varphi_{b}\left(t_{0}\right)}{2}\right)] \end{array}$$

(A13) $$\begin{array}{ccc} \; & =-J_{2}\sin\left(\frac{\varphi_{b}\left(t\right)+\varphi_{b}\left(t_{0}\right)}{2}\right)\sin\left(\gamma_{b}\left(t,t_{0}\right)\right), & \; \end{array}$$

and

(A14) $$\begin{array}{cc} \; & u_{0}\left(t,s\right){\tilde{u}}_{3}\left(s,t_{0}\right)+u_{0}\left(s,t_{0}\right){\tilde{u}}_{3}\left(t,s\right)-{\left(\tilde{u}\left(t,s\right)\times \tilde{u}\left(s,t_{0}\right)\right)}_{3}\\ \; & =-\cos\left(\gamma_{b}\left(t,s\right)\right)\cos\left(\gamma_{b}\left(s,t_{0}\right)\right)\sin\left(\frac{\varphi_{b}\left(t\right)-\varphi_{b}\left(t_{0}\right)}{2}\right)\\ \; & -J_{1}\cos\left(\frac{\varphi_{b}\left(t\right)-\varphi_{b}\left(t_{0}\right)}{2}\right)\left[\cos\left(\gamma_{b}\left(t,s\right)\right)\sin\left(\gamma_{b}\left(s,t_{0}\right)\right)+\sin\left(\gamma_{b}\left(t,s\right)\right)\cos\left(\gamma_{b}\left(s,t_{0}\right)\right)\right]\\ \; & +J_{1}^{2}\sin\left(\gamma_{b}\left(t,s\right)\right)\sin\left(\gamma_{b}\left(s,t_{0}\right)\right)\sin\left(\frac{\varphi_{b}\left(t\right)-\varphi_{b}\left(t_{0}\right)}{2}\right)\\ \; & +J_{2}^{2}\sin\left(\gamma_{b}\left(t,s\right)\right)\sin\left(\gamma_{b}\left(s,t_{0}\right)\right)\sin\left(\frac{\varphi_{b}\left(t\right)-\varphi_{b}\left(t_{0}\right)}{2}\right) \end{array}$$

(A15) $$\begin{array}{cc} \; & =-J_{1}\cos\left(\frac{\varphi_{b}\left(t\right)-\varphi_{b}\left(t_{0}\right)}{2}\right)\sin\left(\gamma_{b}\left(t,t_{0}\right)\right)-\sin\left(\frac{\varphi_{b}\left(t\right)-\varphi_{b}\left(t_{0}\right)}{2}\right)\cos\left(\gamma_{b}\left(t,t_{0}\right)\right). \end{array}$$

The comparison of Equation (A9),(A11),(A13),(A15) with the expressions for functions $u_{0}\left(t\right),\tilde{u}\left(t\right)$ in Theorem 3 proves that the unitary evolution qubit operator $U_{H}\left(t,t_{0}\right),$ specified in Theorem 3, satisfies the cocycle property (70).

This concludes the proof of Theorem 3.

## Appendix B

In this section, we show that the gradient of $K_{3}\left(r\right)$ is given by Equation (50). By Equation (49), we have

(A16) $$\begin{array}{cc} \frac{\partial K_{3}\left(r\right)}{\partial r} & =-i\sqrt{2}\sum_{k=0,1,2}[\frac{r}{{∥r∥}_{R^{8}}}\sin\left(\phi (r)+2\pi k/3\right)\\ \; & +{∥r∥}_{R^{8}}\frac{\partial \phi (r)}{\partial r}\cos\left(\phi (r)+2\pi k/3\right)]\\ \; & \times \exp\left\{-i\sqrt{2}{∥r∥}_{R^{8}}\sin\left(\phi (r)+2\pi k/3\right)\right\}. \end{array}$$

Using further Equation (48), we derive

(A17) $$\frac{\partial \phi (r)}{\partial r}=-\frac{1}{\cos(3\phi (r))}\left(\frac{r}{{∥r∥}_{R^{8}}^{2}}\sin\left(3\phi \left(r\right)\right)+\frac{\sqrt{3}}{2}\frac{1}{{∥r∥}_{R^{8}}^{3}}\frac{\partial }{\partial r}\left(\det(r\cdot \Lambda )\right)\right),$$

where

(A18) $$\begin{array}{cc} \det(r\cdot \Lambda ) & =2(r^{\left(1\right)}r^{\left(4\right)}r^{\left(6\right)}+r^{\left(1\right)}r^{\left(5\right)}r^{\left(7\right)}+r^{\left(2\right)}r^{\left(5\right)}r^{\left(6\right)}-r^{\left(2\right)}r^{\left(4\right)}r^{\left(7\right)})\\ \; & +\frac{1}{\sqrt{3}}r^{\left(8\right)}\left(2{\left(r^{\left(1\right)}\right)}^{2}+2{\left(r^{\left(2\right)}\right)}^{2}+2{\left(r^{\left(3\right)}\right)}^{2}-{\left(r^{\left(4\right)}\right)}^{2}-{\left(r^{\left(5\right)}\right)}^{2}-{\left(r^{\left(6\right)}\right)}^{2}-{\left(r^{\left(7\right)}\right)}^{2}\right)\\ \; & +r^{\left(3\right)}\left({\left(r^{\left(4\right)}\right)}^{2}+{\left(r^{\left(5\right)}\right)}^{2}-{\left(r^{\left(6\right)}\right)}^{2}-{\left(r^{\left(7\right)}\right)}^{2}\right)-\frac{2}{3\sqrt{3}}{\left(r^{\left(8\right)}\right)}^{3}. \end{array}$$

Taking into account that the symmetric structure constants $d_{ijk}$ of SU(3) have the form (see, e.g., Reference [16]):

(A19) $$\begin{array}{cc} d_{146} & =d_{157}=d_{256}=d_{344}=d_{355}=-d_{247}=-d_{366}=-d_{377}=\frac{1}{2},\\ d_{118} & =d_{228}=d_{338}=-d_{888}=-2d_{448}=-2d_{558}=-2d_{668}=-2d_{778}=\frac{1}{\sqrt{3}}, \end{array}$$

we derive

(A20) $$\frac{\partial }{\partial r^{\left(l\right)}}\left(\det(r\cdot \Lambda )\right)=2\sum_{i,j=1}^{8}r^{\left(i\right)}r^{\left(j\right)}d_{ijl}.$$

Hence, Equation (A17) reduces to

(A21) $$\frac{\partial \phi (r)}{\partial r}=-\frac{1}{\cos(3\phi (r))}\left(\frac{r}{{∥r∥}_{R^{8}}^{2}}\sin\left(3\phi \left(r\right)\right)+\sqrt{3}\frac{p(r)}{{∥r∥}_{R^{8}}}\right)$$

and, for Equation (A16), we obtain

(A22) $$\begin{array}{cc} \frac{\partial K_{3}\left(r\right)}{\partial r} & =-i\sqrt{2}\sum_{k=0,1,2}[\frac{r}{{∥r∥}_{R^{8}}}\left(\sin\left(\phi \left(r\right)+2\pi k/3\right)-\sin\left(3\phi \left(r\right)\right)\frac{\cos(\phi (r)+2\pi k/3)}{\cos(3\phi (r))}\right)\\ \; & -\sqrt{3}p\left(r\right)\frac{\cos(\phi (r)+2\pi k/3)}{\cos(3\phi (r))}]\times \exp\left\{-i\sqrt{2}{∥r∥}_{R^{8}}\sin\left(\phi \left(r\right)+2\pi k/3\right)\right\}. \end{array}$$

Noting that, on the right-hand side of Equation (A22), $\cos\left(3\phi (r)\right)=\cos\left(3\left(\phi (r)+2\pi k/3\right)\right)$,

(A23) $$\begin{array}{cc} - & \frac{\cos(\phi (r)+2\pi k/3)}{\cos(3(\phi (r)+2\pi k/3))}=-\frac{1}{4\cos^{2}\left(\phi \left(r\right)+2\pi k/3\right)-3}\\ \; & =\frac{1}{1-2\cos(2(\phi (r)+2\pi k/3))} \end{array}$$

and

(A24) $$\begin{array}{cc} \; & \sin\left(\phi (r)+2\pi k/3\right)-\frac{\sin(3\phi (r))}{\cos(3\phi (r))}\cos\left(\phi (r)+2\pi k/3\right)\\ \; & =-\frac{\sin\left(2(\phi (r)+2\pi k/3)\right)}{\cos(3\phi (r))}=\frac{2\sin(\left(\phi (r)+2\pi k/3\right)}{1-2\cos(\left(2(\phi (r)+2\pi k/3)\right)}, \end{array}$$

for the second and the first terms in the right-hand side of Equation (A22), we come correspondingly to the following expressions:

(A25) $$-i\sqrt{6}p\left(r\right)\sum_{k=0,1,2}\frac{\exp\left\{-i\sqrt{2}{∥r∥}_{R^{8}}\sin\left(\phi \left(r\right)+2\pi k/3\right)\right\}}{1-2\cos(2(\phi (r)+2\pi k/3))}=-i\sqrt{6}F_{1}\left(r\right)p\left(r\right)$$

and

(A26) $$\begin{array}{cc} \; & -i\sqrt{2}\frac{r}{{∥r∥}_{R^{8}}}\sum_{k=0,1,2}\sin\left(\phi (r)+2\pi k/3\right)\frac{\exp\left\{-i\sqrt{2}{∥r∥}_{R^{8}}\sin\left(\phi \left(r\right)+2\pi k/3\right)\right\}}{1-2\cos(\left(2(\phi (r)+2\pi k/3)\right)}\\ \; & =-i\sqrt{6}F_{2}\left(r\right)\frac{r}{{∥r∥}_{R^{8}}}, \end{array}$$

where functions $F_{1}\left(r\right)$ and $F_{2}\left(r\right)$ are given by Equation (51). Relations (A22), (A25) and (A26) prove Equation (50).
